# ‘Saga Stories in health talks’ for health promotion in Swedish child healthcare: results from a cluster-randomised hybrid type 1 effectiveness-implementation study

**DOI:** 10.1186/s12889-025-22786-1

**Published:** 2025-05-02

**Authors:** Maria Henström, Christine Delisle Nyström, Susanne Andermo, Kristin Thomas, Marie Löf

**Affiliations:** 1 Department of Medicine, Huddinge, Karolinska Institutet, Huddinge, 141 83 Sweden; 2 Department of Neurobiology, Care Sciences and Society, Division of Nursing, Karolinska Institutet, 141 83 Huddinge, Sweden; 3 Department of Physical Activity and Health, The Swedish School of Sport and Health Sciences, 114 33 Stockholm, Sweden; 4 Department of Health, Medicine and Caring Sciences, Division of Society and Health, Linköping University, 581 83 Linköping, Sweden

**Keywords:** Child healthcare, Health promotion, Implementation science, Lifestyle behaviours, Parental self-efficacy, Pre-school children, Randomised controlled trial

## Abstract

**Background:**

Early-life interventions are essential for improving public health since lifestyle behaviours are established already in childhood. Child healthcare (CHC) plays a crucial role in this context. The ‘Saga Stories in health talk’ (SSiHT) material includes a flipchart with colourful illustrations to facilitate CHC nurses’ routine health talks with parents and their children, and a hand-out material kit to support healthy lifestyle behaviours in the families. Our aim was to evaluate the effectiveness of the SSiHT intervention at the 5-year routine visit at CHC on parental self-efficacy (primary outcome) and children’s health-related behaviours (secondary outcomes). Implementation aspects for using the SSiHT material in routine practice for health talks at the 5-year visit were simultaneously evaluated.

**Methods:**

A hybrid type 1 effectiveness-implementation design was used to evaluate the intervention in six health regions across Sweden. A cluster-randomised controlled trial was conducted to evaluate effectiveness, where 40 CHC centres (98 nurses) were randomised into two arms: use the SSiHT material in routine care (intervention), or standard care (control). Parents (*n* = 698) of 5-year-olds were recruited. Outcome measures were assessed using digital questionnaires before the health talk (baseline), and two months later (follow-up). Linear mixed-effect models were used to contrast differences in outcomes between groups, in accordance with the study protocol. Acceptability, appropriateness, feasibility, fidelity, and adoption were evaluated using questionnaires and checklists to CHC nurses in the intervention group.

**Results:**

No statistically significant intervention effects were found on parental self-efficacy to promote healthy lifestyle behaviours in their children (-0.61 score on a scale 0–160; *p* = 0.56), nor children’s intake of vegetables, fruits/berries, and sweet drinks or screen time (*p* > 0.05 for all). However, CHC nurses overall reported that the SSiHT material was feasible and appropriate to use in the health talks with families, and they used it in 83% of their health talks.

**Conclusion:**

Although the SSiHT material was well accepted by the CHC nurses, there was no intervention effect on parental self-efficacy (primary outcome) nor health-related behaviours in children when evaluated in Swedish CHC. This warrants further research to better understand how to effectively empower parents through CHC health talks.

**Trial registration:**

Registered 2 February 2022 at Clinicaltrials.gov NCT05237362; https://www.clinicaltrials.gov/study/NCT05237362.

**Supplementary Information:**

The online version contains supplementary material available at 10.1186/s12889-025-22786-1.

## Background

Recent international data on 7,017 children showed that only 14.3% of 3–4-year-olds meet the WHO recommendations for physical activity, screen time and sleep [1]. Also, in the 2024 Generation Pep report [2], self-reported data from a representative sample of the Swedish population showed that only 14% of children aged 4–17 years consume the recommended intake of fruit and vegetables, and 76% do not reach the recommended 60 min of moderate-to-vigorous physical activity per day.

Lifestyle (eating and movement) behaviours are established in early childhood [3]. Parents are key in shaping these behaviours, as their own eating habits and feeding strategies influence their children’s behaviours [4]. A critical factor in this process is parental self-efficacy (PSE), which refers to beliefs and confidence in being able to promote healthy habits in their children, for instance by providing healthy foods, structuring meals, and setting limits [5, 6]. Research has shown that measures of PSE are positively associated with healthier food intake [7–11], higher levels of physical activity [11], and inversely associated with screen time [7] in children. As a determinant for behaviour change [12], PSE is therefore a relevant outcome to assess in effectiveness trials for this target group [5].

Child healthcare (CHC) has been recognised as an important arena for the promotion of healthy lifestyle behaviours [13]. Here, healthcare professionals play an essential role to guide and empower parents in establishing healthy habits in their families [14, 15]. In Sweden, a key component to fulfil this task is ‘health talks’ at routine visits at CHC [16]. The health talk comprises of a conversation between the CHC nurse and the family around health eating and physical activity behaviours in children. However, leading these conversations can be challenging for nurses who perceive certain topics (e.g., food habits, screen time, and child weight) as more sensitive to discuss [17, 18]. Previous research has also reported that health behaviours are infrequently discussed at CHC as most focus is devoted to physical examinations and language development assessments [19]. Moreover, a national survey to map CHC work has shown that a variety of materials are being used in routine health talks in different Swedish regions to inform families about healthy lifestyle behaviours [20], such as the ‘Child-centred Health Dialogue’ (CCHD) [21], ‘Bamse magazine’, and ‘Saga Stories’ (book) [22]. The national CHC guidelines emphasises the importance of CHC’s work being grounded in scientific evidence, and states that elements that are already in use in clinical practice should be scientifically evaluated [15]. To date, only one of these (CCHD) has been scientifically evaluated in a randomised controlled trial and no overall intervention effects were observed [21, 23].

The ‘Saga Stories in Health Talks’ (SSiHT) is material for CHC nurses intended to support them in facilitating their health talks with families. It includes a flipchart with colourful illustrations for the CHC nurse to use in the health talk, as well as hand-out materials for the family: the book ‘Saga Stories: Your amazing body and brain’, fruit- and vegetable bingo, physical activity fortune teller, and the’Pep 24-h day’ poster [24]. In 2021, the material was pilot tested in 11 CHC centres in three Swedish health regions and evaluated through a qualitative study with semi-structured interviews with CHC nurses (*n* = 17) who used SSiHT at the 4-year visit [22]. The CHC nurses found the material extensive, but relevant and useful for the routine health talk, and described how the illustrations helped them involve the children in the health talks. Also, the 5-year visit was deemed as more appropriate for using the material, because of less time constraints and that children have a better ability to concentrate and understand the material at that age [22]. During 2022 we conducted a hybrid type 1 effectiveness-implementation study including a full-scale randomised controlled trial to evaluate the effectiveness and implementation aspects of SSiHT at 5 years of age. This paper reports the main results of the trial according to the study protocol [24].

The specific aims were to:

1. evaluate the effectiveness of the SSiHT intervention on (a) parental self-efficacy to promote healthy lifestyle behaviours in their child (primary outcome); (b) children’s intake of fruit and vegetables as well as sweet drinks (secondary outcomes); and (c) children’s screen time (secondary outcome).
2. evaluate and explore the implementation of SSiHT with regards to acceptability, appropriateness, feasibility, fidelity, adoption, sustainability, and usage.

Sustainability aspects of intervention implementation, explored through semi-structured interviews with CHC nurses, will be reported separately.

## Methods

### Study design

**Fig. 1:**
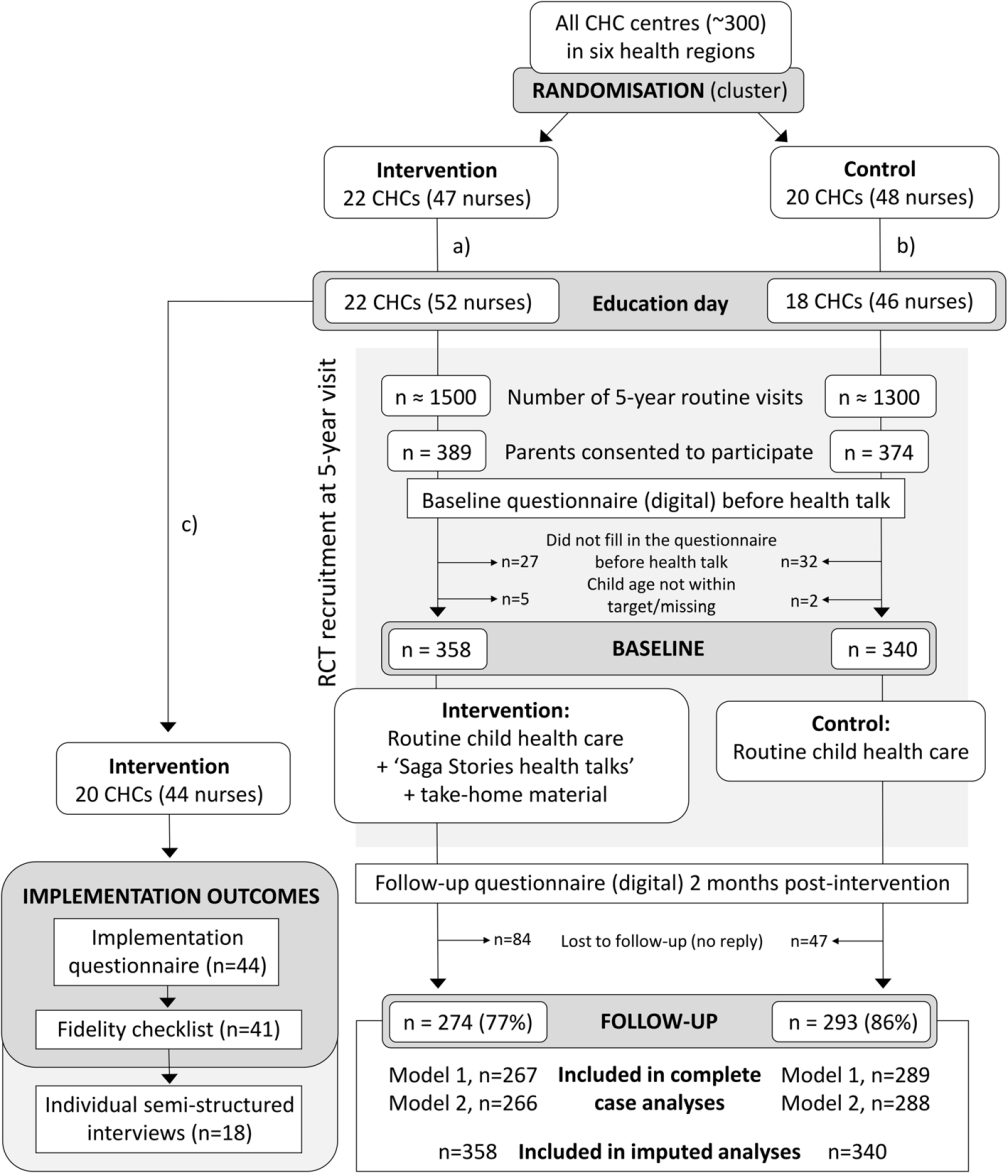
Adapted CONSORT flowchart describing recruitment and data collection. Abbreviations: CHC, child healthcare; RCT, randomised controlled trial. **a** Before the education day but after randomisation, 6 nurses were added from already involved CHC centres, and one other CHC nurse had to withdraw participation (unknown reason). **b** Before the education day but after randomisation, 2 nurses were added from already involved CHC centres, and 4 nurses from 2 different CHC centres had to withdraw participation (reasons: work circumstances and covid- 19-related circumstances). **c** Intervention CHC nurses drop-out during implementation period: lack of time/personnel so had to prioritise other tasks (*n* = 4); lack of time, stressed (*n* = 2); changed workplace (*n* = 1); did not see any 5-year-olds during this period (*n* = 1). No dropouts of CHC nurses in the control group centres

A hybrid type 1 effectiveness-implementation study design was used to enable simultaneous evaluation of both effectiveness (primary focus) and implementation aspects of SSiHT [25]. The study design is described in detail in the study protocol [24] and illustrated using an adapted CONSORT (Consolidated Standards of Reporting Trials) flowchart in Fig. 1. Data collection took place between February and November 2022. The trial is reported according to the CONSORT 2010 statement [26], and the Template for Intervention Description and Replication (TIDieR) [27], Additional files 1–2.

### Participants and baseline procedures

This study was conducted in six health regions across Sweden: Dalarna, Norrbotten, Värmland, Västernorrland, Västra Götaland, and Örebro. All CHC centres (~ 300) in the regions were invited to participate. Nurses who showed interest received detailed written information and were invited to an online meeting before they agreed to participate. Overall, 95 CHC nurses from 42 CHC centres chose to participate in this study. Participating CHC centres were randomly assigned 1:1 to either the intervention group or control group. The cluster-level randomisation was stratified by centre size (1–2 or 3–7 CHC nurses) and geographic region and was performed by a statistician. Prior to data collection, CHC nurses attended a half-day online education on data collection procedures (all centres) and instructions on how to use the intervention material (intervention centres only). Before the education day, eight additional nurses from already involved CHC centres were included, and five nurses had to withdraw their participation with reasons being changed work circumstances (*n* = 1), covid-19 related (*n* = 3) and unknown (*n* = 1). Thus, a total of 40 CHC centres and 98 nurses participated in this study.

At the 5-year routine visit, parents were recruited to the study by their CHC nurse who provided oral and written study information. Eligible parents were those who could read and understand Swedish sufficiently well to provide informed consent and complete the questionnaires. Informed consent was received from all participating parents using a secure digital signature (BankID). They were thereafter automatically presented with the digital baseline questionnaire to fill in during the visit, before the health talk. As CHC nurses used the intervention material in the health talk, neither nurses nor parents were blinded.

### Intervention

SSiHT was developed by Generation Pep, a Swedish non-profit organisation working with health promotion in children and adolescents [28]. It was created based on a request from CHC nurses who currently used the educational children’s book ‘Saga Stories; Your amazing body and brain’ as a hand-out to 5-year-olds but now asked for working materials related to the book to facilitate their health talks. The creation process involved an iterative approach with feedback from CHC nurses, dietitians, paediatricians, and researchers. The material is shown in Additional file 3, and a detailed description is provided in the study protocol [24]. In brief, it consists of 1) a large flip-chart with colourful illustrations on five different health topics: food; physical activity and active play; sedentary behaviour (incl. screen time) and sleep; dental health; and bathroom habits, 2) take-home material intended to promote healthy behaviours at home, and 3) the book `Saga Stories: Your amazing body and brain´ (published in 2017 by Bonnier Carlsen; author Josefin Sundström; illustrator Emma Göthner). The flipchart is used by the CHC nurse during the health talk to facilitate the conversation and involve the child to share his or her experiences and thoughts. In addition to the book, the take-home material includes a physical activity fortune teller and a fruit-and-vegetable bingo, intended to stimulate active play and create curiosity in the child for exploring new foods. Parents were also given a hand-out that illustrates an ideal 24-h distribution of time for a 5-year-old child to spend on sleeping, physical activity, playing, and using screens.

### Control group

Nurses at control CHC centres were asked to continue with the 5-year visits as usual. According to the National Handbook for Child Health Services, standard care at the 5-year health visit includes a general health talk around healthy lifestyle behaviours [29]. Participating families in the control group received standard care but without the SSiHT material or similar. However, after the follow-up questionnaire, the family was offered a ’Saga Stories’ book.

### Effectiveness outcome measures

An electronic questionnaire was filled in by the parent who accompanied the child, at baseline and again at follow-up 2 months later. The baseline questionnaire included a brief section with demographic questions regarding their own age, sex, weight, height, and socioeconomic status, as well as their child’s sex, date of birth and country of birth. Children’s weight and height was measured using standardised procedures at the CHC visit and recorded in the questionnaire. A secure online survey tool (esMaker) was used and the link to the follow-up questionnaire was sent by SMS to the parent by the researchers, with a maximum of two reminders.

#### *Parental *self*-efficacy*

The primary outcome was PSE regarding the parent’s own perceived ability to promote healthy lifestyle behaviours in their child. To assess PSE, the validated “Parental Self-Efficacy for Promoting Healthy Physical Activity and Dietary Behaviours in Children Scale” (PSEPAD) Questionnaire was used [5]. It includes 16 questions which cover PSE for promoting healthy dietary behaviours in children (7 questions), promoting healthy physical activity behaviours (3 questions), and for limit-setting of unhealthy dietary or physical activity behaviours (6 questions). Each question was answered by rating their PSE on a scale from 0 (“Not at all”) to 10 (“To a very large extent”). For the analyses, a total PSE score (maximum 160) was created and treated as a continuous variable. Separate analyses for each of the three PSE sub-scores were also performed.

#### Health* behaviours*

Secondary outcomes in this study were children’s health behaviours. A modified version of the Swedish National Board of Health and Welfare’s survey regarding health behaviours [30] to assess key indicators of children’s dietary intake was used [31]. Three questions were included which asked parents to estimate their child’s intake of 1) vegetables including root vegetables, 2) fruits and berries, and 3) sweet drinks. The amounts were assessed as the average number of portions per day (one portion roughly corresponding to a child’s hand full of vegetables, or 1 normal-sized fruit or 1 dl berries/fruit), and parents were asked to report the usual intake over the past month. Children’s intake of sweet drinks was assessed as average frequency per day, and included soft drinks, juice, cordial, energy drinks, chocolate drinks, fruit soup, sweetened yoghurt, and similar. The reported number of portions were converted to grams per day using standardised food item weights from the national food database provided by the Swedish Food Agency [32], as previously done [31]. Children’s screen time, i.e., the average amount of time spent watching smartphones/tablets/TV/computer was estimated by parents in minutes per day on a typical day when the child was at home i.e., not at preschool.

### Implementation outcomes

Implementation evaluation took place in the 22 CHC centres in the intervention group, where all participating nurses were new to using the full SSiHT material i.e., flipchart and hand-outs. Throughout the intervention period, the nurses in both groups had access to an online folder with project instructions and the education day recording. They also received monthly newsletters from Generation Pep. In addition, nurses in the intervention group had copies of the intervention material in an online folder and received invitations to online discussion forums to share experiences (total three 1 h-sessions). Implementation outcomes have been thoroughly described in the study protocol [24]. In brief, a Swedish version of the validated questionnaire developed by Weiner et al. [33] was used to assess *acceptability, appropriateness,* and *feasibility*. This was filled in by the CHC nurses three months after implementation. All nurses also filled in a checklist after each 5-year routine visit, taking notes on whether they used the material or not, what parts of the flipchart they used, and what take-home materials were given to the family. These checklists were collected by the researchers at the end of the period and used to assess *fidelity* and *adoption*. Furthermore, parental perceptions on their child’s involvement in the health talks, as well as of their *usage* of the take-home material, were assessed using intervention-specific questions in the follow-up questionnaire filled in by parents in the intervention group. A purposeful sample of the CHC nurses were also interviewed to investigate what key factors influence *implementation sustainability*; those results will be reported in a separate manuscript.

### Sample size

With 80% power, a minimum of 368 parent–child dyads (184 per arm) were required to detect a four-point difference in total PSE score between intervention and control group. As described previously [24], the power calculation was based on a linear random-intercept model which included a centre-specific normal random intercept to account for within-centre stochastic dependence of the data. One-thousand pseudo-random Monte-Carlo samples were generated under an assumed intra-centre correlation (ICC) of 0.03 and specified group differences, with coefficients and regression residual variances based on unpublished data on PSE from another health promotion trial (MINISTOP 1.0) [34]. Based on previous research [34, 35], a 20% drop-out rate was assumed, and we aimed to recruit 450 parent–child dyads (225 per group).

### Statistical analyses

The statistical analyses followed the study protocol [24] and were performed using R (v.4.2.1, R Core Team, Vienna, Austria, 2022). Questionnaires and checklists were summarised using descriptive statistics, and linear mixed-effect models were used to contrast differences in outcomes between intervention and control at follow-up. The crude model was adjusted for measured outcome at baseline, and CHC centre (random effect) to account for clustering. The second model was adjusted also for child age (continuous) and sex (dichotomous). All analyses were intention-to-treat with primary analysis performed on completers only. We estimated the intraclass correlation coefficient (ICC), which is the proportion of variance in the outcome (PSE) explained by the clustering, using the icc() R function on the unconditional model. Attrition analyses were performed, and the data was deemed to be missing at random. Sensitivity analyses were run by imputing missing data (multivariate imputations by chained equations, creating 200 datasets with 30 iterations using the “mice” R package) [36]. Furthermore, interaction analyses were conducted with regards to parental age (continuous), education status (University vs. no University), and body mass index (BMI) (continuous), by including an interaction term in the adjusted complete-case model. *P*-values of < 0.05 were considered statistically significant.

## Results

### Participants

Recruitment and data collection is illustrated in the CONSORT flowchart in Fig. 1. During the 7.5 months recruitment period, approximately 2800 five-year routine visits occurred at the 40 CHC centres (based on expected number of visits per year estimated by the nurses). At those visits, a total of 698 accompanying parents at intervention (*n* = 358) and control (*n* = 340) CHC centres agreed to participate in the study and filled in the baseline questionnaire. Table 1 shows baseline characteristics of participating parents and children. Most parents were mothers (74%), about half (52%) had a university education, and the majority (91%) were born in Sweden. Among the children, 65 (9.6%) were classified according to ISO-BMI cutoffs [37] as having underweight, 540 (79.4%) normal weight, and 75 (11.0%) overweight or obesity. There were no major differences between the intervention and control group in the outcome variables at baseline. Two months after the health visit, 274 (77%) of participants in the intervention group and 293 (86%) in the control group replied to the follow-up questionnaire (Fig. 1). Only eleven participants had missing data in the primary outcome at follow-up and thus a total of 556 subjects were included in the complete case analyses.

**Table 1:** Baseline characteristics of participating parents and children at the 5-year visit

	**All (*n*** **= 698)**		**Intervention (*n* = 358)**		**Control (*n* = 340)**	
	**N**	**% or mean (SD)**	**N**	**% or mean (SD)**	**N**	**% or mean (SD)**
**Parents**						
Gender						
Female	513	74.1%	260	73.0%	253	75.3%
Male	179	25.9%	96	27.0%	83	24.7%
Age (years)	693	36.0 (5.2)	357	37.2 (5.3)	336	35.8 (5.0)
Education						
University	361	52.2%	190	53.5%	171	50.9%
No university	330	47.8%	165	46.5%	165	49.1%
Country of birth						
Sweden	632	91.1%	316	88.5%	316	93.8%
Other	62	8.9%	41	11.5%	18	5.4%
BMI (kg/m^2^)	687	25.3 (4.6)	353	25.2 (4.6)	334	25.3 (4.6)
PSE total score^1^	689	124.6 (17.0)	354	123.3 (17.2)	335	126.0 (16.8)
**Children**						
Gender						
Girls	346	49.9%	175	49.2%	171	50.7%
Boys	347	50.1%	181	50.8%	166	49.3%
Age at baseline	698	5.1 (0.2)	340	5.1 (0.1)	358	5.2 (0.2)
Country of birth						
Sweden	688	98.9%	353	98.6%	335	99.1%
Other	8	1.1%	5	1.4%	3	0.9%
BMI (kg/m^2^)	685	15.7 (1.4)	353	15.7 (1.3)	332	15.6 (1.5)
BMI z-score^2^	680	0.12 (0.94)	351	0.13 (0.89)	329	0.10 (0.99)
BMI classification^3^						
Thinness	65	9.6%	30	10.0%	35	10.6%
Normal weight	540	79.4%	281	80.1%	259	78.7%
Overweight	62	9.1%	36	10.3%	26	7.9%
Obesity	13	1.9%	4	1.1%	9	2.7%
Vegetable intake (g/day)	689	40 (18)	354	40 (18)	335	40 (19)
Fruit/berry intake (g/day)	686	152 (65)	351	149 (64)	335	155 (67)
Sweet drinks intake (g/day)	685	73 (89)	353	70 (81)	332	76 (98)
Screen time (min/day)	689	121 (62)	354	122 (63)	335	119 (61)

*Abbreviations*: *SD* standard deviation, *BMI* Body Mass Index, *PSE* Parental Self-efficacy

^1^ Total score calculated as the sum of all 16 PSE questions (0–10) creating a maximum score of 160

^2^ BMI standard deviation scores (z-scores) calculated using the extended international (IOTF) age and sex specific BMI cut-offs as described by Cole TJ & Lobstein T. Extended international (IOTF) body mass index cut-offs for thinness, overweight and obesity. Pediatr Obes. 2012;7(4):284–94

^3^ BMI classification according to cut-offs by Cole & Lobstein: Thinness, ISO-BMI < 18.5 kg/m^2^; Normal weight, ISO-BMI = 18.5–24.9 kg/m^2^; Overweight, ISO-BMI = 25.0 − 29.9 kg/m^2^; Obesity, ISO-BMI > = 30.0 kg/m^2^

### Effectiveness of the intervention

We observed no statistically significant intervention effects on the primary nor secondary outcomes (Table 2). Reported PSE was on average slightly lower at follow-up than at baseline in both groups, but there was no statistically significant difference between intervention and control in the complete-case analysis (−0.92; 95% CI −2.86 to 1.00; *p* = 0.35: adjusted model). PSE sub-scales were also analysed (Table 3) and no significant effects on PSE for promoting children’s healthy dietary behaviours (− 0.78; 95% CI −1.79 to 0.21; *p* = 0.12), physical activity behaviours (−0.46; 95% CI −0.96 to 0.04; *p* = 0.070), or limit-setting (0.17; 95% CI −0.83 to 1.16; *p* = 0.74) were observed. The ICC was 0.002, suggesting very little of the variance in the PSE variable was due to CHC clusters. Regarding secondary outcomes, no evidence of an intervention effect was seen on children’s vegetable intake (−0.4; 95% CI −2.6 to 1.9; *p* = 0.74), fruit/berry intake (1.2; 95% CI −7.8 to 10.2; *p* = 0.79), sweet drinks (7.2; 95% CI −8.7 to 22.4; *p* = 0.35), or screen time (2.5; 95% CI − 7.1 to 11.7; *p* = 0.56) (all adjusted model).

**Table 2:** Intervention effect results on primary and secondary outcomes

	**Descriptive data follow-up**		**Complete cases analysis**						**Imputed data analysis**	
	**Intervention** *n* = 267	**Control** *n* = 289	**Crude**^1^			**Adjusted**^2^			**Adjusted**^3^ *n* = 698	
	**mean (SD)**	**mean (SD)**	**N**	**Coefficient (95% CI)**	**P**	**N**	**Coefficient (95% CI)**	**P**	**Coefficient (95% CI)**	**P**
*Primary outcome*										
**PSE total score**^4^	121.6 (17.2)	124.3 (16.6)	556	−1.19 (−3.18 to 0.80)	0.23	554	−0.92 (−2.86 to 1.00)	0.35	−0.61 (−2.73 to 1.45)	0.56
*Secondary outcomes*										
**Vegetable intake (g/day)**	39 (18)	39 (17)	552	−0.3 (−2.6 to 1.9)	0.76	550	−0.4 (−2.6 to 1.9)	0.74	0.5 (−2.3 to 3.4)	0.75
**Fruit/berry intake (g/day)**	143 (64)	148 (63)	549	−1.1 (−7.8 to 9.9)	0.81	547	1.2 (−7.8 to 10.2)	0.79	0.1 (−9.1 to 9.2)	0.98
**Sweet drinks (g/day)**	74 (87)	71 (78)	547	7.1 (−8.9 to 22.4)	0.37	545	7.2 (−8.7 to 22.4)	0.35	11.1 (−8.8 to 30.5)	0.26
**Screen time (min/day)**	127 (62)	126 (63)	552	2.0 (−7.6 to 11.3)	0.68	550	2.5 (−7.1 to 11.7)	0.56	1.3 (−9.8 to 12.2)	0.81

*Abbreviations*: *PSE* parental self-efficacy, *SD* standard deviation, *CI* confidence interval, *P* *p*-value

^1^ Crude linear mixed-effect model adjusted only for baseline value and CHC centre (random intercept). Coefficient can be interpreted as the mean difference between groups at follow-up (intervention – control)

^2^ Linear mixed-effect model adjusted for baseline value, CHC centre (random intercept), child age and sex

^3^ Imputed analysis was adjusted for baseline value, CHC centre (random intercept), child age and sex. *N* = 698 (intervention *n* = 358, control *n* = 340)

^4^ Total score calculated as the sum of all 16 PSE questions (0–10) creating a maximum score of 160

**Table 3:** Intervention effect results on parental self-efficacy subscales

	**Descriptive data** (n = 544)^1^				**Complete-case analysis**^2^	
	**Intervention** n = 266		**Control** n = 288			
	**mean (SD)**		**mean (SD)**		**Coefficient (95% CI)**	**P**
*PSE subscales*	Baseline	Follow-up	Baseline	Follow-up		
**PSE dietary behaviours**^3^	57.1 (7.9)	56.1 (8.3)	57.3 (8.2)	57.1 (7.7)	−0.78 (−1.79 to 0.21)	0.12
**PSE physical activity**^4^	24.3 (4.1)	23.8 (4.2)	24.5 (4.0)	24.4 (3.7)	−0.46 (−0.96 to 0.04)	0.070
**PSE limit-setting**^5^	42.6 (7.9)	41.8 (7.6)	44.4 (7.9)	42.8 (8.0)	0.17 (−0.83 to 1.16)	0.74

*Abbreviations*: *PSE* parental self-efficacy, *SD* standard deviation, *CI* confidence interval, *P p*-value

^1^ Descriptive data for the 544 subjects with complete PSE data at baseline and follow-up, and data for adjusted variables i.e., child age and sex

^2^ Linear mixed-effect model adjusted for baseline value, CHC centre (random intercept), child age and sex. Coefficient can be interpreted as the mean difference between groups at follow-up (intervention – control)

^3^ Parental self-efficacy for promoting healthy dietary behaviours in children, assessed with 7 questions (0–10), maximum score 70

^4^ Parental self-efficacy for promoting healthy physical activity behaviours in children, assessed with 3 questions (0–10), maximum score 30

^5^ Parental self-efficacy for limit-setting of unhealthy dietary or physical activity behaviours in children, assessed with 6 questions (0–10), maximum score 60

Similar results were observed in the sensitivity analyses, where imputed data (n = 698) showed no statistically significant differences between groups for PSE (−0.61; 95% CI −2.73 to 1.45; *p* = 0.56) or the secondary outcomes, see Table 2. Interaction analyses were performed and no evidence of the intervention effects on PSE being moderated by parents’ age, education, nor BMI were observed. With regards to secondary outcomes, significant p-values were detected for interaction between parents’ education level and children’s fruit/berry intake (*p* = 0.024), but stratified analysis did not show any significant effects within education sub-groups.

### Implementation outcomes

Nurses at the 22 CHC centres allocated to the intervention arm received SSiHT material to implement at all 5-year routine visits. During the evaluation period, eight CHC nurses had to withdraw their participation in the project (most common reason was lack of time). All remaining 44 nurses returned the implementation questionnaire and 41 returned their checklist, representing 19 of 20 CHC centres in the implementation arm, see Fig. 1.

#### Acceptability, appropriateness, and feasibility

**Fig. 2:**
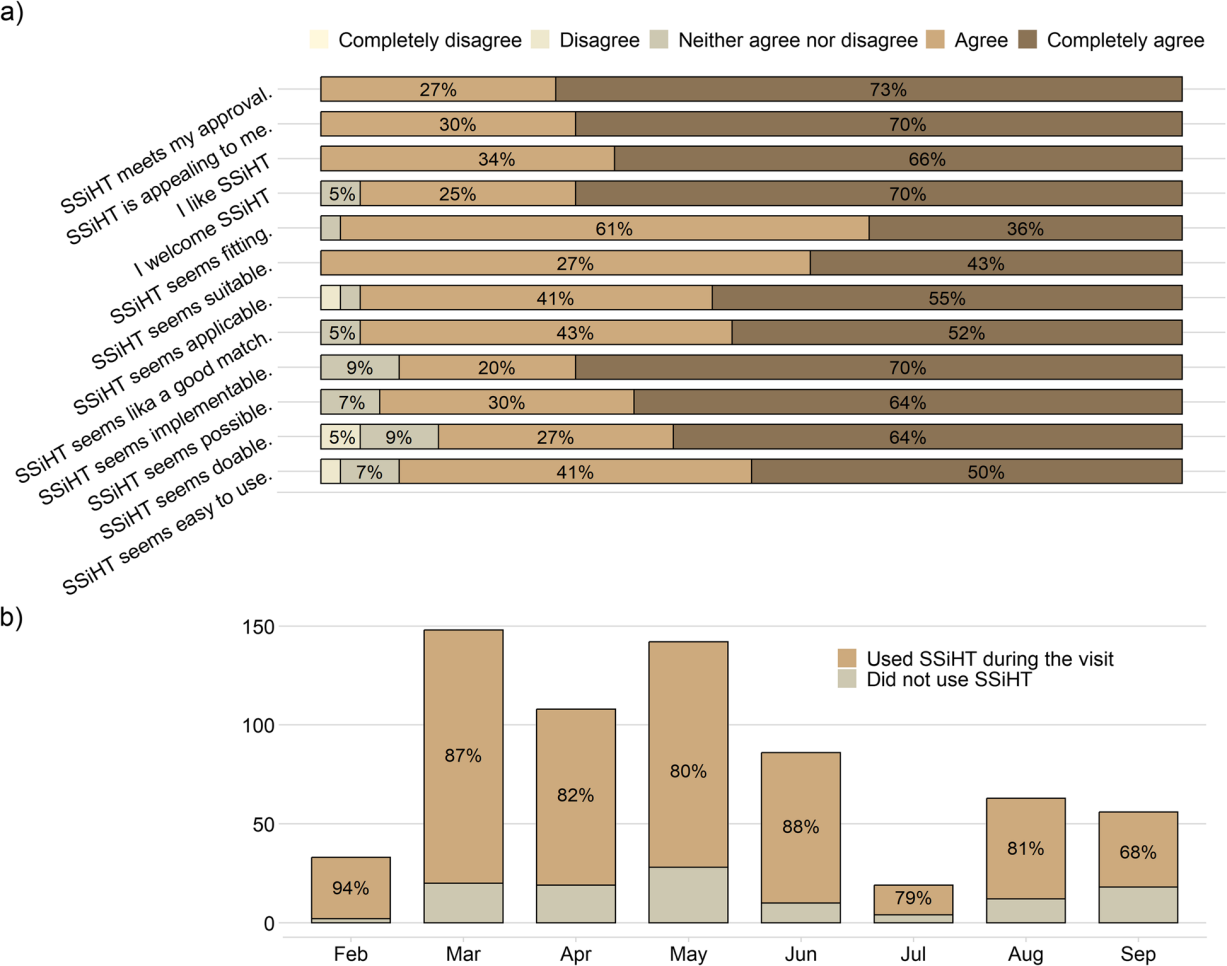
Implementation aspects of ‘Saga Stories in health talks’ (SSiHT) based on information from nurses. **a** Participating child healthcare nurses’ (*n* = 44) reported answers to the Acceptability of Intervention Measure (AIM; top 4 questions), Intervention Appropriateness Measure (IAM; middle 4 questions), and Feasibility of Intervention Measure (FIM; lower 4 questions) regarding the SSiHT material. Data from the questionnaire by Weiner et al. [33] filled in 3 months after initiating implementation. **b** Adoption of SSiHT at the 5-year routine visits, shown as percentage of all conducted health talks per month where SSiHT was used. Data from the evaluation period Feb 3rd until Sep 22nd 2022, recorded in checklists by 41 nurses at 19 child healthcare centres allocated to the intervention arm. Overall adoption rate for the entire 7.5-months period was 83%

The responses to the implementation questionnaire [33] are summarised in Fig. 2a. Overall, the CHC nurses agreed or completely agreed to acceptability aspects of implementing SSiHT in their practice. Most nurses also believed the material was appropriate, and that implementation was feasible, although slightly more nurses answered neutrally to the latter.

#### Fidelity and adoption

Figure 2b shows the adoption of SSiHT during the evaluation period, as recorded in the CHC nurses’ checklists. A total of 655 five-year visits were reported and the intervention material was used in 542 (83%) of the visits. The most used section of the flipchart was food (91% of the SSiHT health talks), followed by physical activity and active play (86%), sedentary behaviour (incl. screen time) and sleep (62%), dental health (56%), and bathroom habits (53%). After the health talk, almost all families received the ‘Saga Stories’ book (98%), while the other take home-materials were given to the following proportion of families: physical activity fortune teller (90%), the fruit-and-vegetable bingo (86%), and the “Pep” 24-h day poster (71%). In a few SSiHT health talks (20 of the 542), CHC nurses had used the book instead of the flipchart. Common reasons for *not* using the SSiHT material were: lack of time at the visit; language barriers; had to prioritise other aspects; or not feasible because the child had a neurodevelopmental disorder, was unfocused, or tired.

#### Child involvement in the health talk and usage of the take-home material

Most of the 261 parents who answered the feedback questions reported that their child had been either partly involved/engaged in the conversation (*n* = 124; 48%) or fully involved and engaged (*n* = 96; 37%). Another 38 children (15%) were a bit involved, whereas 3 (1%) did not participate at all. Ninety-one percent of the families in the intervention group reported that they had received the book (*n* = 241). The majority had used it at home since the visit two months ago; 130 parents (54%) had read it together with their child once or twice, and 98 (41%) at least three times. The physical activity fortune teller had been played with by 175 (75%) of children who had received it, but usage of the fruit and vegetable bingo was lower (49% of children), see Additional file 4.

## Discussion

### Main findings

In this hybrid type 1 cluster-randomised controlled study, we evaluated the effectiveness as well as implementation aspects of the health promotion material SSiHT to be used at the 5-year routine visit in Swedish CHC. No statistically significant intervention effects were detected on PSE to promote healthy lifestyle behaviours in their children (primary outcome), nor children’s intake of vegetables, fruits/berries, or sweet drinks, or screen time (secondary outcomes). However, the CHC nurses reported the material to be feasible and appropriate for health conversations with families. They reported using it in the majority of their health talks (83%).

### Comparison with previous work

The primary outcome in this study was carefully chosen. Considering the low dose of this intervention, detectable effects on physical health indicators such as children’s BMI z-scores were not expected. Instead, we hypothesised that health talks with the SSiHT material, which aims to highlight and encourage positive health behaviours in a supportive and non-judgemental way, could have an impact on PSE; however, this was not the case in this study. These results are in agreement with a previous evaluation of another illustration-based material used in health talks with slightly younger children, where no overall effect on PSE was observed [21]. In contrast, significant effects on PSE have been shown for other interventions in the CHC context targeting the same health behaviours. For instance, the PRIMROSE intervention (repeated motivation interviewing [MI] sessions with parents during the first 4 years) [38] as well as the MINISTOP app (a 6-month digital parental program) [31] had statistically significant effects on PSE for promoting healthy dietary behaviours. The average item score of 7.8 (on a scale of 0–10) at baseline in our study may leave little room for improvement. However, this level is similar to the above-mentioned cohorts, such as MINISTOP (average 7.5 per item at baseline) [31]. Other possible explanations for the lack of effects in the current trial may be differences in intervention intensity, length, and content. Both PRIMROSE and MINISTOP are more comprehensive programs. PRIMROSE applied MI techniques, MINISTOP incorporated a series of behaviour change techniques [31, 39] and both were grounded in social cognitive behaviour theory [12].

Moreover, no effects on children’s health behaviours were observed in the current study. For comparison, the recently published evaluation of the MINISTOP intervention demonstrated positive effects on children’s intakes of sweet and savoury treats and sweet drinks, as well as screen time [31]. Furthermore, the PRIMROSE trial showed small but statistically significant intervention effects on eating habits at age 4 years [35]. The CCHD trial, which is another illustration-based material used for child-centred health conversations in Sweden, included similar outcome measures [40]; however, those results have not yet been reported so we cannot compare our findings. Internationally, a recent Cochrane review concluded that child-feeding practice interventions and multicomponent interventions for children up to 5 years or age can have a small but positive short-term (< 12 months) impact on children’s vegetable intake [41]. Also, there is a growing body of evidence that obesity prevention interventions delivered earlier in life (first 2 years) to parents can promote healthy child-feeding practices and improve children’s eating- and movement behaviours [42, 43]. In the current study, the low intervention dose and/or relatively little focus on sedentary behaviours and screen time (covered in 62% of health talks as reported by CHC nurses) may explain the lack of effect on children's lifestyle behaviours. It is also important to note that in primary prevention, interventions provided as universal care (i.e., to all irrespective of health status), such as SSiHT, are typically complex and broad and may not lead to detectable effects on BMI or health status; however, reasonable outcomes could be improved self-efficacy, knowledge or health literacy. Self-efficacy is an important construct for motivation to change [12] and is also a target measure in CHC primary prevention work (parent ‘empowerment’) [15].

In our study, CHC nurses appreciated working with the SSiHT material, as evident from their high usage rate and reported agreement with feasibility, appropriateness, and acceptability aspects (Fig. 2). This is in line with findings from the qualitative SSiHT pilot study [22] and another CCHD evaluation [44], where CHC nurses often welcomed these types of material to be used for involving the child in talks around healthy habits. This is a positive finding. However, it was not used in slightly more than one in six health talks, and further investigation is needed to understand the underlying reasons and how adoption can be improved.

Taken together, child-centred health talks with the SSiHT material at 5-year visits were appreciated by CHC nurses. However, our results indicate that this material did not strengthen parents to stimulate behaviour change in their families. Considering the limited time available at CHC visits, digital interventions such as the 6-month MINISTOP parental program [31, 34] could be a way of providing evidence-based support between visits at CHC.

### Strengths and limitations

The hybrid design used in this study is the main strength, as it allows for simultaneous evaluation of both effectiveness and implementation aspects in real-world circumstances; these are both important to consider before translating into routine care [25]. Other strengths include the randomised controlled design and the large sample size which enabled well-powered analyses. CHC centres included in this study spanned six health regions across Sweden, representing both low and high socioeconomic areas, and a potential clustering effect was accounted for in both power calculation and analyses. Centres were randomised with both centre size and location in consideration and the low ICC of 0.002 suggests that very little of the variance in PSE was due to the clustering. Furthermore, we used a validated instrument to assess PSE (primary outcome) [5, 45]. Finally, the SSiHT material was developed in an iterative process using feedback from CHC nurses and other stakeholders and was pilot tested for its usability before being evaluated in the current study [22].

This study also had some limitations. First, although the health talks were structured in accordance with the provided intervention material, there might have been minor variation in how the nurses spoke to the families. However, this applies to both groups. Furthermore, because the intervention material and questionnaires were only available in Swedish, only Swedish-speaking families could participate. This limits the generalisability of the results, considering that around one in four 5-year-olds in Sweden have foreign born parent(s) [46]. It is important that health promotion and supporting initiatives are accessible to all families, irrespective of background, to counteract current inequalities in health. However, more research is needed on cultural adaptations before these types of material can be used with non-Swedish speaking families, addressing important aspects such as limited literacy to access information [47]. Consideration of how to use the material with interpreters is also important, as this can be a significant barrier for CHC nurses to convey health messages as intended [18, 48].

### Implications for child health care and future research

This was a hybrid type 1 effectiveness-implementation study where the primary aim was to evaluate effectiveness. We did not observe any effects on PSE (primary outcome) or lifestyle behaviours. However, as the nurses appreciated the material, our findings clearly motivate further research. Firstly, it is relevant to note that the SSiHT concept was developed to enable involvement of the child, based on the Convention on the Rights of the Child [49]. This became law in Sweden in 2020 and resulted in an expectation on CHC nurses to more clearly acknowledge the child’s perspective during health visits [15]. It was not part of our study protocol [24] to assess how the children perceived this information and their involvement in the health talk, but this is clearly an area where more knowledge is needed. Previous research has shown that children at 4 years may not interpret health talk messages and illustrations as intended [50], and children as young as 3–5 years could potentially conceptualise a link between weight gain and eating patterns [51]. Having conversations with parents and children together requires an ability to balance the dialogue with the child vs. parents; something that can be challenging, especially when overweight is identified [44, 52]. CHC nurses have also previously raised concerns around causing harm to children that are present in the room when weight issues are being discussed [53]. In contrast, our qualitative pilot data suggests that some of the nurses who used SSiHT at routine visits managed to reach the parent “through the child” [22]. Clearly, more research is required regarding which health conversations [19], to what degree, and how, children should be involved. Finally, further studies should investigate whether additional or modified CHC visits may improve effectiveness on PSE or children’s lifestyle behaviours.

## Conclusions

In this hybrid cluster-randomised controlled trial, we evaluated the effectiveness of SSiHT as well as implementation aspects for its use in health talks at the 5-year routine CHC visit. Our findings suggest that the SSiHT material was appreciated by CHC nurses; however, despite a well-powered and rigorous study design, no intervention effects on PSE, nor health-related behaviours in children, were observed. Further research is warranted to better understand how it can be used to support parents in the health talk.

## Supplementary Information

Additional File 1. Consolidated Standards of Reporting Trials (CONSORT) checklist for reporting on randomised trial

Additional File 2. The Template for Intervention Description and Replication (TIDieR) checklist for reporting of interventions

Additional File 3. The‘Saga Stories in health talks’ intervention material

Additional File 4. Parents’ reported child involvement in the health talk and usage of the ‘Saga Stories in health talks’ material

## Data Availability

The datasets generated and analysed during the current study are available from the corresponding author on reasonable request.

## Abbreviations

BMI: Body mass index
CHC: Child healthcare
ICC: Intraclass correlation coefficient
PSE: Parental self-efficacy
SSiHT: Saga Stories in Health Talks
