# Supplementary material for: ‘Saga Stories in health talks’ for health promotion in Swedish child healthcare: results from a cluster-randomised hybrid type 1 effectiveness-implementation study
Source: BMC Public Health. 2025 May 2;25:1637. doi: 10.1186/s12889-025-22786-1 (PMC12046779; doi:10.1186/s12889-025-22786-1)
Supplement: Supplementary file 3 — Additional File 3. The‘Saga Stories in health talks’ intervention material [file 12889_2025_22786_MOESM3_ESM.pdf]

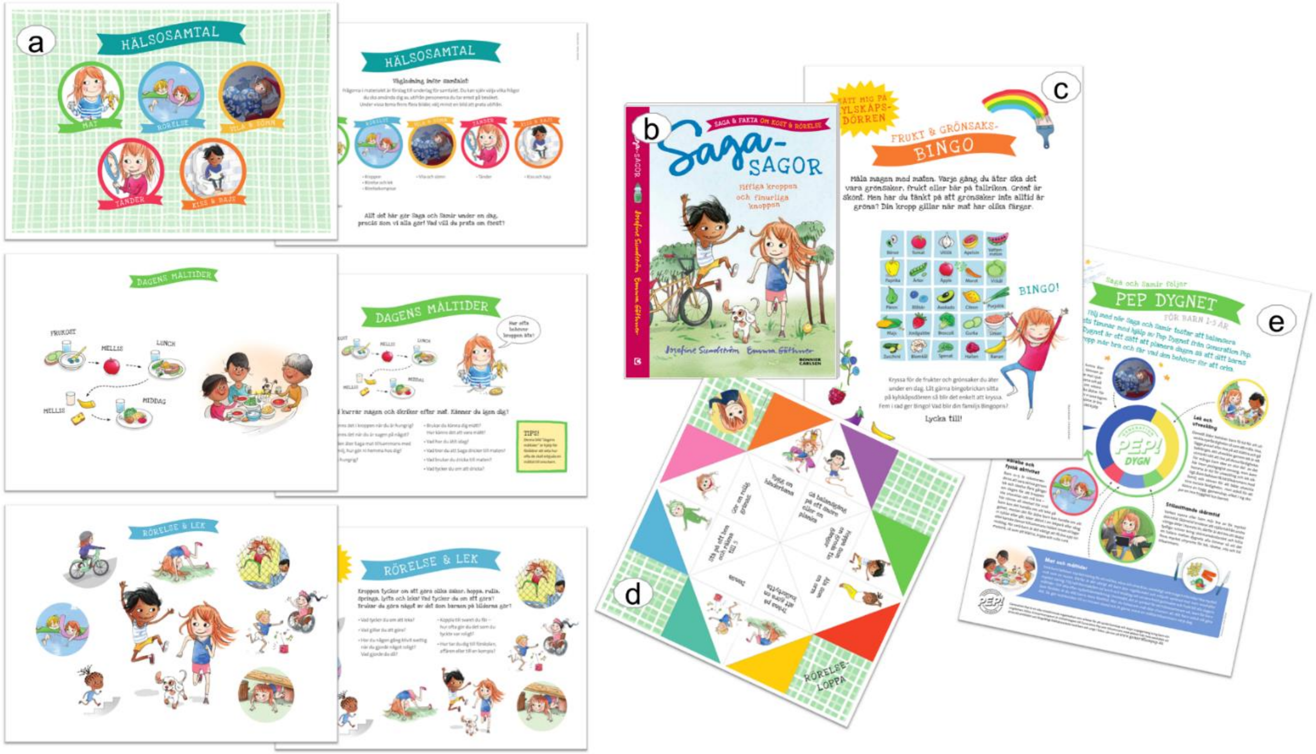

Additional file 3. The 'Saga Stories in health talks' intervention material.

a) Front page and examples from food and physical activity sections of the flipchart used by the nurse in the health talk. On the left are the pages that are shown to the families, and on the right are the back of the same pages where the nurse is provided with helpful information and suggestions of questions to ask the child. The flipchart contains a total of 15 pages (front- and back) covering the topics: food; physical activity and active play; sedentary behaviour and sleep; dental health; and bathroom habits. b) The fictional but educational book `Saga Stories: Your amazing body and brain´ published in 2017 by Generation Pep and Josefin Sundström (Bonnier Carlsen publisher). c) Fruit- and vegetable bingo to inspire children to try new foods. d) Physical activity fortune teller, to fold and play with to inspire active play. e) The 'Pep' 24-h day poster, given out to parents to inform them on how an ideal distribution of time spending on sleeping, being physically active, playing, and using screens around the age of 5 years. All illustrations are made by Emma Göthner.
