# Supplementary material for: ‘Saga Stories in health talks’ for health promotion in Swedish child healthcare: results from a cluster-randomised hybrid type 1 effectiveness-implementation study
Source: BMC Public Health. 2025 May 2;25:1637. doi: 10.1186/s12889-025-22786-1 (PMC12046779; doi:10.1186/s12889-025-22786-1)
Supplement: Supplementary file 4 — Additional File 4. Parents’ reported child involvement in the health talk and usage of the ‘Saga Stories in health talks’ material [file 12889_2025_22786_MOESM4_ESM.pdf]

Additional File 4. Parents' reported child involvement in the health talk and usage of the 'Saga Stories in health talks' material.

**Child involvement**	My child was fully involved and engaged in the conversation	My child was involved/engaged in the conversation	My child was a bit involved/engaged	My child did not want to/could not participate in the conversation	*n*[1]
How involved was your child in the conversation around the pictures?	96 (37%)	124 (48%)	38 (15%)	3 (1%)	261

**Usage**[2]	Received it	Used it 3 or more times	Used it 1-2 times	Did not use it	*NA*
How often have you looked in or read the 'Saga Stories' **book** together, since the CHC visit?	241 (91%)	98 (41%)	130 (54%)	11 (5%)	2
How often have you/your child used the **physical activity fortune teller**, since the CHC visit?	233 (88%)	92 (39%)	83 (36%)	58 (25%)	0
How often have you/your child used the **fruit and vegetable bingo**, since the CHC visit?	218 (82%)	61 (28%)	45 (21%)	111 (51%)	1
		Used it		Did not use it	
Have you used the **"Pep" 24-h day poster**, and if so, how?	101 (38%)	68 (67%)<br>47 parents read it, 18 put it up at home (e.g., on the fridge), and/or 15 talked about it with their child		33 (22%)	0

[1] *n* refers to the number of participants who answered the related question. A total of 265 participants in the intervention group filled in the feedback section in the follow-up questionnaire. Nine participants answered the follow-up questionnaire but none of the feedback questions so are therefore not included in this table.

[2] In the digital questionnaire, the usage questions were only asked to those parents who reported receiving that take-home material in an initial question (with numbers from that question reported as "Received it").

CHC, Child healthcare.
